# Topical Use of Tacrolimus in Corneal and Ocular Surface Pathologies: A Systematic Review

**DOI:** 10.3390/jcm14155347

**Published:** 2025-07-29

**Authors:** Georgios Katonis, Argyrios Tzamalis, Ioannis Tsinopoulos, Nikolaos Ziakas

**Affiliations:** 1 Postgraduate Master Program “Ocular Surgery”, School of Medicine, Aristotle University of Thessaloniki, University Campus, 54124 Thessaloniki, Greece; gkatonis@auth.gr (G.K.); itsinop@auth.gr (I.T.); ngziakas@auth.gr (N.Z.); 2 2nd Department of Ophthalmology, Aristotle University of Thessaloniki, Papageorgiou General Hospital, 56429 Thessaloniki, Greece

**Keywords:** tacrolimus, ophthalmology, topical administration, vernal keratoconjunctivitis, corneal transplantation

## Abstract

**Background/Objectives**: Tacrolimus, an immunosuppressant, is increasingly used topically in ophthalmology, particularly for conditions like vernal keratoconjunctivitis and post-keratoplasty rejection prophylaxis. This systematic review aims to evaluate the efficacy and safety of topical tacrolimus in these ocular conditions. **Methods**: A thorough search was conducted in PubMed and Cochrane Library for relevant studies published up to 16 March 2025. Studies were eligible for inclusion if they were randomized controlled trials investigating topical tacrolimus in human ocular disease, were published in English, and reported clearly defined outcomes. Exclusion criteria included non-randomized studies, animal studies, systemic treatments, non-English publications, and studies lacking clearly reported outcomes. Data regarding study design, patient demographics, intervention details, and outcomes were extracted and analyzed. The Cochrane risk-of-bias tool (RoB 2.0) was used to assess the risk of bias. **Results**: A total of 10 studies met the inclusion criteria, were retrieved, and were categorized as not highly biased after the risk-of-bias assessment. These studies were included in the systematic review, where a qualitative analysis took place. Our analysis revealed that the topical use of tacrolimus showed promising results, as it improved clinical signs and symptoms in most patients. In half of the studies, tacrolimus demonstrated superior efficacy compared to the control group, while in the remaining studies, it showed equivalent efficacy. Adverse effects, such as a burning sensation, were noted in 7/10 studies but were generally mild. The methodologies were somewhat heterogeneous, and some studies had small sample sizes. **Conclusions**: Topical tacrolimus shows promising effects in managing various ocular surface diseases. While randomized controlled trials provide evidence, further research with larger sample sizes is necessary to solidify its efficacy and safety profile compared to other immunosuppressants.

## 1. Introduction

Tacrolimus (also known as FK-506) is a macrolide immunosuppressant, discovered in Japan in 1984, that is isolated from *Streptomyces tsukubaensis* and is 10–100 times more potent than cyclosporine A, both in vivo and in vitro [1]. Its mechanism of action is similar to that of cyclosporine; both belong to the same macrolide family. Tacrolimus can inhibit T-cell proliferation and T-cell-mediated production of inflammatory mediators. This is accomplished through the inhibition of calcineurin, a molecule vital for the transcription of interleukins IL2 and IL4 [2,3]. Other immunomodulatory properties include the inhibition of histamine release from mast cells and basophils and the suppression of prostaglandin synthesis [4,5].

Because of these immunomodulatory and immunosuppressant properties, tacrolimus is widely used in ophthalmology for the control of autoimmune diseases like uveitis, prevention of rejection after corneal transplantation, management of ocular manifestations in graft-versus-host disease, dry eye disease, and allergic conditions such as atopic keratoconjunctivitis and vernal keratoconjunctivitis [3]. Given that its systemic administration may be risky due to various neurological, cardiovascular, and metabolic side effects, its topical usage has gained ground in recent decades [6,7]. Its greater efficacy compared to steroids and much fewer ocular side effects have made its use very practical [3]. However, despite its efficacy, there is a lack of comprehensive systematic reviews evaluating the effectiveness and safety profile of tacrolimus and its topical ophthalmic use.

This systematic review aims to address all well-organized studies of topical tacrolimus in ophthalmology by thoroughly examining the existing literature and conducting a qualitative analysis of its benefits, implications, and limitations. Additionally, the review aims to highlight research gaps to pave the way for further primary research.

## 2. Methods

This systematic review was conducted in accordance with the PRISMA (Preferred Reporting Items for Systematic Reviews and Meta-Analyses) 2020 guidelines. The completed PRISMA 2020 checklist is available in the Supplementary Materials.

### 2.1. Ethics and Registration

This study is a systematic review of previously published data and does not involve any new studies with human participants performed by the authors. Therefore, ethical approval and informed consent were not required. The study was not registered in PROSPERO or any other registry.

### 2.2. Search Strategy

A comprehensive literature search was conducted through the databases PubMed and Cochrane Library for relevant studies. Relevant medical terms, such as “tacrolimus,” “eye”, “allergic keratoconjunctivitis”, and others, were combined using Boolean logic operations. The exact text used in the search for both databases was as follows: tacrolimus AND (eye OR ophthalmic OR ophthalmology OR ocular OR conjunctivitis OR keratoconjunctivitis OR keratoplasty OR dry eye OR keratitis OR sicca OR uveitis). The database search was limited to publications up to 16 March 2025. In the PubMed database, the search was applied to All Fields, while in the Cochrane Library database, it was applied to All Text.

The full electronic search strategies for each database are provided in Supplementary Table S1.

### 2.3. Criteria for Literature Selection

The review was guided by the PICO framework:

Population—Human patients diagnosed with ocular surface or adnexal diseases for which the topical use of tacrolimus is applicable. Intervention—Topical tacrolimus administered as eye drops or ointment in varying concentrations. Comparator—Placebo, other topical treatments, or, in some cases, surgical procedures. Outcome—The efficacy of tacrolimus in treating ocular disease, either as a standalone measure or in comparison with other treatments, as well as the safety profile and reported adverse effects.

Studies were selected based on predefined inclusion and exclusion criteria.

Inclusion Criteria

The following criteria were used for article selection:

1. Randomized controlled trials (RCTs);
2. Written in English;
3. Human trials;
4. Topical administration of the drug;
5. Well-defined primary and secondary outcomes;
6. Clear primary and/or secondary outcome data.

Exclusion Criteria

The following criteria were used to exclude articles:

1. Non-RCTs, reviews, observational studies, case reports, and editorial publications;
2. Articles with only published abstracts;
3. Non-English language articles;
4. Non-human trials;
5. Systemic administration of the drug;
6. Ambiguous, unmentioned, or absent primary and/or secondary outcomes;
7. Studies with unclear and poorly presented data.

### 2.4. Study Selection

All references extracted through the literature search were imported into EndNote Library (EndNote 21.2). This application was used to detect and remove duplicates with the same title. Two authors then conducted a careful and independent title and abstract examination of the remaining articles. Any disagreements between reviewers during the study selection phase were resolved through discussion and consensus. During this stage, articles that did not meet the inclusion criteria, met the exclusion criteria, or were irrelevant were removed. Additional duplicates with slightly different titles were also identified and removed. The authors retrieved the full texts of the remaining articles. The articles with available full texts were then reviewed for eligibility and the quality of the available data.

### 2.5. Data Collection and Extraction

Two authors worked independently to obtain the necessary data. Any disagreements between reviewers during the data extraction phase were resolved through discussion and consensus. After a thorough examination of each trial, the desired data were extracted. The extracted data included the first author’s name, year of publication, country of origin, basic study design, sample size, mean age of subgroups, medical condition examined, intervention drugs, drug concentrations, galenic form of each drug, frequency of administration, total duration of therapy, timing of re-evaluations, stated and measured primary and secondary outcomes, and brief conclusions about the drugs tested. No statistical effect measures were applied, as no meta-analysis was performed. Instead, a qualitative synthesis was conducted, summarizing and comparing study findings narratively.

### 2.6. Risk-of-Bias Assessment

The risk of bias within the included studies was evaluated by an author using the Cochrane risk-of-bias tool (RoB 2.0) [8]. No disagreements arose during this procedure, as all assessments of risk of bias were performed by a single author. In the assessment of bias across the included studies, a meticulous evaluation of each study took place, using the five domains of the Cochrane risk-of-bias tool, to ensure a comprehensive analysis. More specifically, Domain 1 addresses bias arising from the randomization process, Domain 2 evaluates bias due to deviations from the intended intervention, Domain 3 addresses bias related to missing outcome data, Domain 4 examines bias in the measurement of outcomes, and lastly, Domain 5 considers bias in the selection of reported results. Publication bias and selective reporting were assessed qualitatively by determining the availability of study protocols and verifying whether all pre-specified outcomes were reported. Any discrepancies between reported outcomes and study conclusions were noted. The certainty of evidence was appraised narratively, taking into account study quality, risk of bias, consistency of findings, and the directness of evidence. Given the absence of a formal meta-analysis, tools such as GRADE were not applied.

### 2.7. Synthesis Methods

Studies were included in the synthesis if they met the predefined eligibility criteria regarding population, intervention, outcomes, and study design. Extracted data were systematically tabulated to facilitate comparison of study characteristics, interventions, and reported outcomes. As no statistical synthesis was performed, the data were presented in their original extracted form without requiring transformation or conversion. Missing data, when encountered, were documented; however, no imputation methods were applied. Findings from individual studies were summarized in tabular format, detailing study design, sample size, intervention characteristics, and key results. A narrative synthesis was undertaken to provide contextual interpretation of the findings across studies. The qualitative synthesis was structured around thematic analysis of study outcomes, with comparisons drawn based on clinical effectiveness, safety profiles, and overall conclusions regarding the use of topical tacrolimus in ophthalmology. Heterogeneity among the included studies was explored narratively by examining variations in study design, patient populations, intervention protocols, outcome measures, and reported conclusions. No statistical assessments of heterogeneity were conducted. Given the qualitative nature of the synthesis, formal sensitivity analyses were not performed. However, differences in study quality and methodological design were taken into account when interpreting the findings.

## 3. Results

### 3.1. Search Results

**Figure 1:**
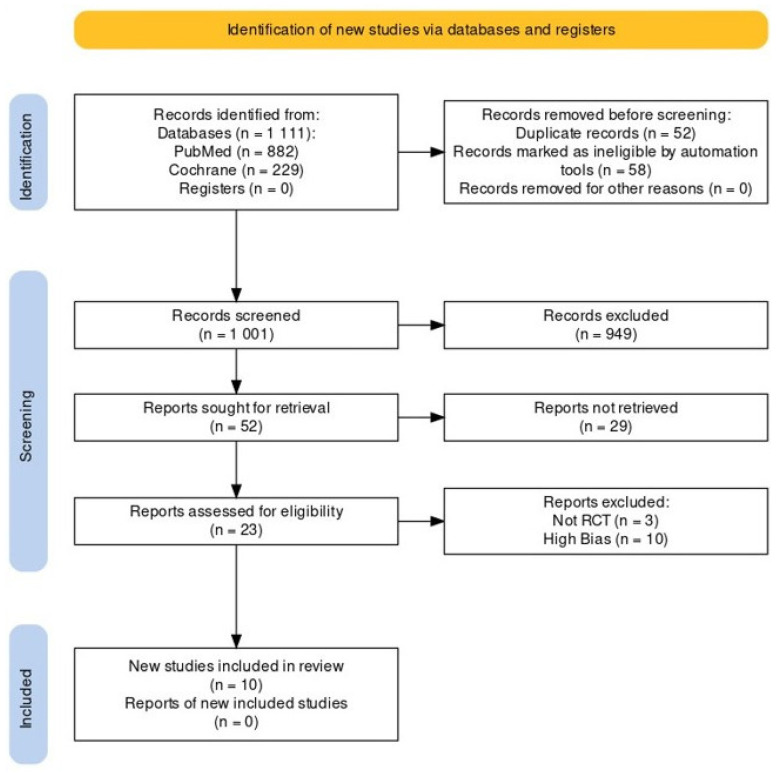
PRISMA 2020 flow diagram of the study selection process.

A total of 1111 articles were retrieved from the database search, including 882 articles from PubMed and 229 articles from the Cochrane Library. After automated removal of duplicates (n = 52) and some ineligible articles (n = 58), 1001 articles proceeded to title and abstract screening. Of these, 52 advanced to retrieval, during which the full text was found for 23 articles. Three of them were neither mentioned as RCTs nor contained RCT characteristics. Data were extracted from the remaining 20 studies, which were then assessed for risk of bias. Ultimately, 10 studies were included in the review. A PRISMA 2020 flow chart was created to illustrate the elimination process [9]. Figure 1 shows the flow chart of literature screening for this systematic review.

### 3.2. Basic Data of the Included Studies

Data were extracted from all 20 studies assessed for risk of bias. The data extraction did not include three studies that lacked clear RCT characteristics. From the final ten studies included, five studies investigated the treatment of vernal keratoconjunctivitis (VKC) with tacrolimus [10,11,12,13,14], one study was about dry eye secondary to Sjogren syndrome [15], one study talked about the place of tacrolimus in the treatment of ocular graft-versus-host disease (GVHD) [16], two studies were about adenoviral corneal subepithelial infiltrates (SEIs) [17,18], and finally, one study investigated the effectiveness of tacrolimus in acute endothelial rejection after penetrating keratoplasty (PKP) [19]. Overall, the total patient size was 377. More specifically, there were 131 patients with VKC, 60 patients with dry eye secondary to Sjogren syndrome, 40 with ocular GVHD, 115 with adenoviral corneal SEIs, and 31 with acute endothelial graft rejection after PKP. The reported data came from different countries: Italy (n = 1), Iran (n = 2), Brazil (n = 2), Thailand (n = 1), Egypt (n = 2), USA (n = 1), and India (n = 1).

For each included study, extracted data were systematically summarized in structured tables. These tables detail study characteristics, intervention protocols, outcome measures, and key findings, among others. Given that no statistical synthesis was performed, effect estimates, confidence intervals, and summary statistics were not applicable. Instead, results were analyzed descriptively, focusing on trends in clinical effectiveness, safety outcomes, and reported conclusions.

All the data of the included studies mentioned in the Methods Section are shown in Table 1 and Table 2.

### 3.3. Assessment of Risk of Bias in the Included Studies

Five different domains of bias were examined in each of the ten studies. Out of the twenty studies, six had high bias arising from the randomization process, two studies had bias due to deviations from intended interventions, one study had bias due to missing outcome data, no study was highly biased in the measurement of outcomes, and finally, five studies showed bias in the selection of reported results. Some studies exhibited high bias in more than one domain.

**Figure 2:**
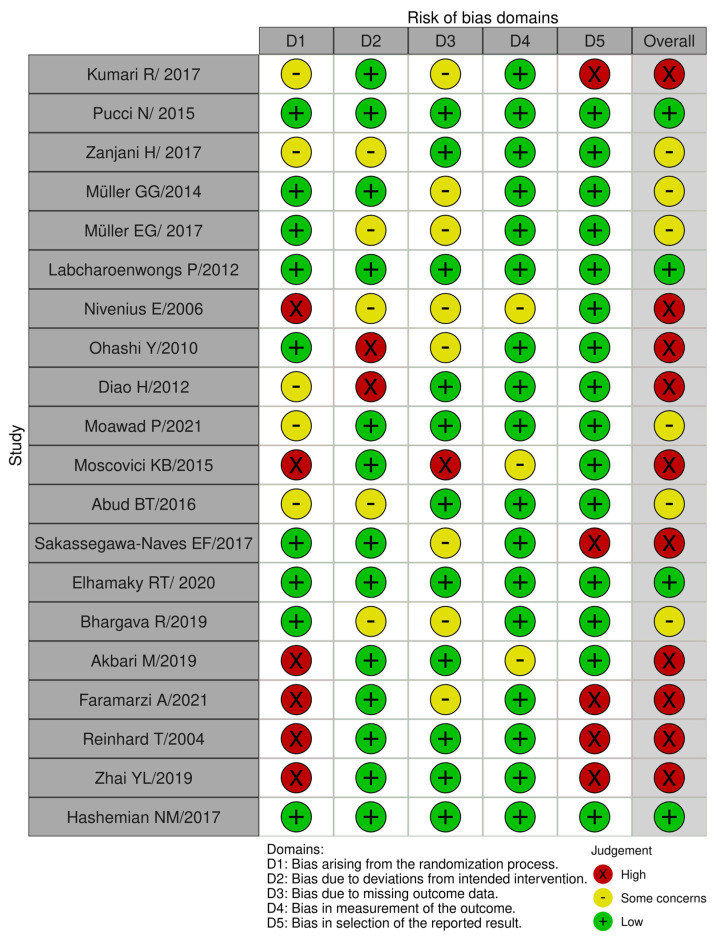
Risk-of-bias assessment #1 [10,11,12,13,14,15,16,17,18,19,20,21,22,23,24,25,26,27,28,29].

**Figure 3:**
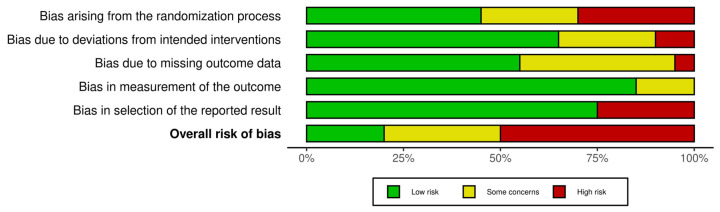
Risk-of-bias assessment #2.

Studies with overall high bias were not included in the systematic review (n = 10). Studies with overall low bias or bias with some concerns were included in the review (n = 10). The conclusions regarding the quality of each study’s methods and the presence of any of the aforementioned types of biases are summarized in Figure 2 and Figure 3.

### 3.4. Summary of Findings and Qualitative Analysis of Results

i. Vernal Keratoconjunctivitis (VKC)

In this systematic review, five randomized controlled trials were examined to assess the therapeutic efficacy of topical tacrolimus (TAC) for vernal keratoconjunctivitis (VKC) at varying concentrations (0.005% to 0.1%) and in different formulations (eye drops versus ointment). These studies included comparisons with standard treatments such as cyclosporine, sodium cromoglycate, and interferon-A 2b, targeting patient populations with cyclosporine-resistant or refractory VKC. The synthesized findings below provide a comparative analysis of TAC’s effectiveness in improving clinical symptoms and signs, its safety profile, and its impact on patients’ quality of life.

While some studies focused on total scores, others provided detailed analyses of individual signs and symptoms. Specifically, two studies [10,14] based their conclusions on the total symptom and sign scores. In contrast, other studies [11,13] presented measurements of each sign and symptom in tables, noting significant differences before and after treatment. Zanjani et al. [11] compared the total scores of each group, whereas Müller et al. [13] did not. Finally, Müller et al. [12] displayed figures of each sign and symptom score but compared only the total score variations between the two groups.

With regard to symptoms, the total symptom score in TAC groups significantly improved in both studies that measured it [10,14]. Itching, photophobia, and tearing improved in two other studies [11,13], while foreign body sensation improved only in Zanjani et al. [11]. The symptom figures in Müller et al. [12] showed improvement in every symptom but were not significant compared to the control group.

Regarding clinical signs, the total sign score in TAC groups significantly improved in both studies that measured it [10,14]. Conjunctival hyperemia and limbal inflammation and hypertrophy improved in both subsequent studies [11,13]. Additionally, Zanjani et al. [11] showed improvement in the Trantas dot sign and corneal punctate epitheliopathy, while Müller et al. [13] did not show improvement in upper tarsal papillae, keratitis, or discharge with the use of tacrolimus. The sign figures in Müller et al. [12] showed improvement for every sign but were not significant compared to the control group, except for conjunctival hyperemia, which worsened in the control group.

As far as safety is concerned, Labcharoenwongs et al. [14] showed non-significant side effects in the TAC group (*p* > 0.05), while two other studies [12,13] presented patients with a burning sensation. Zanjani et al. [11] had one patient with redness and itching from administration that needed steroids. Finally, Pucci et al. [10] presented the most side effects, as all their patients experienced burning and some stinging and pain on administration, but these side effects tended to improve. In the end, only a few patients experienced burning and stinging.

Quality of life was assessed only in the study of Pucci et al. [10], in which it showed significant improvement.

Clinical impression assessment and self-assessment were performed only in the study of Müller et al. [12]. The results varied within each group, but there were no differences between the groups.

In comparison with the control groups, Müller et al. [12] showed no significant difference between the TAC group and the TAC-with-additional-olopatadine group, except for its unique significant improvement in conjunctival hyperemia due to olopatadine’s side effect. This shows that the addition of olopatadine to the treatment regimen was useless. In the study of Müller et al. [13], TAC had significantly higher efficacy in improving many signs and symptoms compared to the control, sodium cromoglycate, which shows TAC’s superiority. In Zanjani et al. [11]’s results, no significant difference between tacrolimus and interferon was noted, as both seemed effective and safe. In their study, Pucci et al. [10] concluded that tacrolimus is effective in cases of cyclosporine-resistant VKC, where cyclosporine (CYC) has no place. However, in cases of common VKC, Labcharoenwongs et al. [14]’s trial showed that neither CYC nor TAC is superior to the other, even though the total sign score was significantly reduced compared to baseline only in the tacrolimus group.

Tacrolimus was used in different concentrations and frequencies in each study. Two studies [10,14] used 0.1% TAC, the former three times daily and the latter twice daily. In both studies, tacrolimus treatment was significantly effective. Two other studies [12,13] used 0.03% TAC, with the former (twice daily) concluding that the addition of an antihistamine agent was useless, while the latter (three times daily) concluded that tacrolimus was superior to sodium cromoglycate. Finally, Zanjani et al. [11] used 0.005% TAC and concluded that it was significantly effective in reducing signs and symptoms. This indicates that, regardless of the concentration or frequency of administration, tacrolimus was effective in patients with this condition.

Tacrolimus was administered as eye drops in three VKC studies [10,11,13] and as an ointment in two others [12,14]. In all three studies utilizing eye drops, tacrolimus demonstrated efficacy. The control groups in these studies were also treated with eye drops: tacrolimus showed comparable efficacy to interferon-alpha 2b in one study [11] and was superior to cyclosporine A and sodium cromoglycate in the other two [10,13]. In the two studies where tacrolimus was administered as an ointment, its effectiveness was similar to that of the control groups. One study included olopatadine eye drops in addition to the standard treatment in the control group [12], while the other included only cyclosporine eye drops in the control group [14]. Thus, tacrolimus in either form was not compared to an ointment drug. Tacrolimus was significantly effective in both of its formulations. However, in two-thirds of cases where it was administered as eye drops, it demonstrated superior efficacy compared to the control group. Conversely, when used as an ointment, it did not show greater efficacy but rather comparable effectiveness to the control group.

In all five studies on the treatment of VKC with tacrolimus, a consistent pattern of effectiveness in improving sign and symptom scores with varying concentrations and frequencies of TAC administration was observed. The studies collectively underscore TAC’s efficacy in managing VKC symptoms and signs, with minimal and manageable side effects reported across different patient cohorts.

ii. Dry eye secondary to Sjögren syndrome

The Moawad et al. study [15] highlights topical tacrolimus (TAC) as a viable option for managing Sjögren syndrome-related dry eye, with outcomes comparable to cyclosporine A (CYC) in reducing artificial tear dependence and enhancing ocular surface health over six months. Tacrolimus 0.03% was administered as eye drops, prepared by dissolving tacrolimus powder into a solution until the desired concentration was achieved. Key evaluations were performed at baseline (day 0), day 90 (3 months), and day 180 (6 months). Both groups were assessed using the Ocular Surface Disease Index (OSDI), tear break-up time (TBUT), SICCA score, van Bijsterveldt score, Schirmer test, frequency of artificial tear use per day, and meibum quality and expressibility. Although neither treatment significantly improved meibomian gland function or tear production (as measured by the Schirmer test), both demonstrated meaningful improvements in ocular surface stability and reduced reliance on artificial tears. No statistically significant differences were observed between the two groups. These findings support the potential of TAC as an alternative to CYC, particularly in cases where CYC is unsuitable, and suggest broader applicability for TAC in the management of autoimmune-related dry eye conditions.

iii. Ocular GVHD (Graft-Versus-Host-Disease)

In their double-blinded RCT, Abud et al. [16] evaluated the efficacy of tacrolimus (TAC) compared to methylprednisolone in patients with ocular manifestations of graft-versus-host disease (GVHD). Tacrolimus was administered as eye drops at a concentration of 0.05%, twice daily for 10 weeks. The eye drops were prepared by processing an intravenous tacrolimus solution (Prograf). Patients were assessed at baseline, after 5 weeks, and at the end of treatment (10 weeks). Outcome measures included Ocular Surface Disease Index (OSDI) scores, corneal fluorescein staining, tear break-up time (TBUT), Schirmer test scores, and the expression levels of HLA-DR and ICAM-1 on the ocular surface. While total discomfort scores showed no significant differences between the two groups, patients receiving TAC reported a higher incidence of burning sensations, a common side effect. However, a key advantage of TAC was its lack of impact on intraocular pressure (IOP), which significantly increased in the methylprednisolone group. Both treatments effectively reduced corneal fluorescein staining scores, but TAC demonstrated superior efficacy (*p* = 0.01). Furthermore, TAC significantly improved mean tear break-up time after 10 weeks, indicating enhanced ocular surface stability. Flow cytometric analysis revealed that both treatments reduced inflammatory markers (HLA-DR and ICAM-1), yet TAC may be the safer option for patients due to its favorable side-effect profile and greater effectiveness in managing symptoms of GVHD-related dry eye disease.

iv. Adenoviral corneal subepithelial infiltrates (SEIs)

Two randomized controlled trials about SEIs were included in this systematic review. Elhamaky et al. [17] compared 0.03% tacrolimus (TAC) ointment (Protopic), administered once daily for 2–6 months, with transepithelial phototherapeutic keratectomy (Te-PTK). Evaluations were conducted at baseline, after 1 week, and then at 1, 3, 6, and 12 months. To assess SEI reduction during treatment, a comprehensive examination was performed at each visit, including the Ocular Surface Disease Index (OSDI) questionnaire, slit-lamp photography, and corneal tomography with Pentacam, which recorded corneal densitometry and higher-order aberrations (HOAs). The authors observed significant reductions in mean corneal densitometry and HOAs in both treatment groups, along with notable improvements in best-corrected visual acuity (BCVA) and OSDI scores. There was no statistically significant difference in any of the measured outcomes between the Te-PTK and TAC groups. The persistence of corneal SEIs was significantly lower in both the Te-PTK and TAC groups than in the control group after 1 year. Bhargava et al. [18], in a double-blind RCT, compared 0.03% TAC ointment (TALIMUS-LS), administered twice daily for 6 months, with dexamethasone (DEXA). Evaluations were performed monthly for intraocular pressure (IOP) and SEI scores and at 1, 3, and 6 months for potential side effects. SEI improvement and symptom relief were assessed using slit-lamp biomicroscopy, the Dry Eye Scoring System (DESS) questionnaire, and visual acuity. The authors concluded that TAC resulted in greater improvement in SEI scores, BCVA, and symptom scores compared to DEXA. Moreover, TAC did not cause IOP elevation, unlike DEXA, although mild side effects such as burning and foreign body sensation were reported.

Overall, both studies support the effectiveness of TAC in improving SEI scores and visual outcomes. TAC demonstrated a more favorable side-effect profile than DEXA, primarily due to the absence of IOP elevation. Both studies used 0.03% tacrolimus ointment, although they differed in their methods of SEI assessment. While both used slit-lamp biomicroscopy, different symptom scoring systems were employed (OSDI vs. DESS), and only Elhamaky et al. incorporated corneal topography using Pentacam.

v. Acute endothelial graft rejection after PKP

In Hashemian et al. [19], tacrolimus (TAC) was tested as an adjuvant to prednisolone in patients with acute endothelial graft rejection following penetrating keratoplasty (PKP). TAC was administered as eye drops at a concentration of 0.05%, prepared by diluting an intravenous formulation (Prograf) with a balanced salt solution. The drops were instilled four times daily until graft rejection reversal, after which a maintenance dose of 0.01% TAC was used four times daily for three months. Patients were evaluated on day 1, on day 3, at week 1, and then every 3–5 days until reversal. After reversal, follow-up assessments were conducted every two weeks for two months, monthly for three months, and every three months thereafter. The outcomes assessed included (1) rejection reversal (defined as the disappearance of specific rejection signs) or failure to reverse (defined as treatment failure); (2) time to rejection reversal (the interval between treatment initiation and clinical reversal); and (3) recurrence of rejection following a successful reversal (reappearance of rejection signs). The rejection signs were corneal edema, keratic precipitates, rejection line, anterior chamber cells and flare, and decreased graft clarity. While initial rejection reversal rates were not significantly different between groups, TAC demonstrated added effectiveness in reducing the time to rejection reversal when baseline differences were accounted for. Furthermore, TAC significantly lowered the recurrence rate of rejection episodes compared to prednisolone alone.

These findings suggest that TAC may enhance the speed and durability of rejection management in PKP patients, supporting its potential as a valuable addition to corticosteroid therapy, particularly for patients at high risk of recurrent rejection.

Table 3 shows a brief review of all 10 studies included and the key findings of each about the efficacy of tacrolimus in comparison to the other therapeutical approaches.

Table 4 shows a brief review of all 10 studies included and the side effects that each group faced during the trial.

## 4. Discussion

Topical tacrolimus has been approved by the Food and Drug Administration for the treatment of moderate to severe atopic dermatitis [30]. Although tacrolimus ointments are intended for dermatologic use, numerous reports document their topical application for inflammatory ocular diseases. This is evident here, as three studies included in this review utilized 0.03% tacrolimus ointment as an off-label treatment for eye conditions [12,17,18], alongside one study that employed a 0.1% tacrolimus ointment [14]. Additionally, various other forms of tacrolimus, such as eye drops in several different concentrations, are used off-label in many studies included in this review and are prepared by hospital or laboratory pharmacists [10,11,13,15,16,19].

In four of the studies where tacrolimus was administered as eye drops, the drops were prepared by diluting tacrolimus solution intended for intravenous use (Prograf; Astellas Pharma Inc., Tokyo, Japan) with balanced salt solution until the desired concentration was achieved [10,11,16,19]. One other study did not mention the preparation method for the eye drops, only stating that they were obtained from Ophthalmos, Brazil [13]. Finally, another study prepared the eye drops by dissolving tacrolimus powder in a specific mixture [15].

Regarding tacrolimus administered as an ointment, two studies utilized Protopic 0.03% tacrolimus ointment (Fujisawa Healthcare Inc. and Astellas Pharma Tech Co.) [12,17], one study employed TALIMUS-LS 0.03% tacrolimus ointment [18], and another study prepared a 0.1% tacrolimus ointment by mixing oral tablets of tacrolimus (Prograf; Fujisawa) with an ophthalmic base [14].

In this systematic review, we meticulously selected only randomized controlled trials (RCTs) to assess the efficacy and safety of topical tacrolimus across a range of ophthalmic conditions, including vernal keratoconjunctivitis (VKC), secondary dry eye disease, ocular graft-versus-host disease (GVHD), adenoviral corneal infiltrates, and endothelial graft rejection. Several pertinent studies were reviewed but ultimately excluded from our analysis due to methodological constraints or their non-RCT design. Previous reviews have also explored the safety and/or efficacy of tacrolimus in some of these conditions. These studies are discussed below to acknowledge their contributions; however, they do not influence the conclusions drawn from our review.

Rasmussen et al. [31] conducted a systematic review and meta-analysis that demonstrated statistically significant improvements in VKC symptoms with tacrolimus, particularly in terms of hyperemia and tearing. Nonetheless, their review encompassed a wide array of treatments beyond tacrolimus, with tacrolimus being only one of several agents assessed. This broad scope may have diluted the specific insights pertaining to tacrolimus alone. As such, while the study highlighted the efficacy of various treatments, its findings on tacrolimus were not the central focus.

Zhao et al. [32] performed a meta-analysis dedicated to VKC and reported favorable outcomes for tacrolimus concerning objective sign scores and subjective symptom scores. However, their analysis was confined to VKC and did not extend to other ophthalmic conditions where tacrolimus could be applied. Furthermore, Zhao et al.’s meta-analysis considered only two outcome measures, thereby limiting the overall evidence base regarding the efficacy and safety of tacrolimus.

Sun et al. [33] provided significant evidence regarding the effectiveness of topical tacrolimus in preventing immune rejection in high-risk penetrating keratoplasty (PKP) patients. Their findings indicated that tacrolimus was more effective compared to topical cyclosporine A.

Abudou et al. [34] reviewed the prophylaxis of graft rejection post-PKP, comparing tacrolimus with topical steroids. Although their study reported that all participants maintained graft survival, it was rated low in quality due to a considerable risk of bias. Consequently, while this study offers relevant insights, it was not utilized to substantiate our review findings due to its methodological limitations.

In summary, while the studies mentioned contribute valuable context to the understanding of tacrolimus in ophthalmic conditions, they are acknowledged here but do not impact the conclusions of our review. Our findings are based on high-quality RCT evidence to ensure the robustness and reliability of the results.

After carefully examining significant and insignificant results from the studies, we summarized the key findings. This review aims to evaluate the safety and efficacy of tacrolimus by synthesizing these findings. The results indicate that tacrolimus is as safe and effective as many established therapies, significantly improving several measured outcomes with manageable side effects. Tacrolimus was superior to the control group in five studies [10,13,16,18,19] and equally effective in comparison with other treatments in five other trials [11,12,14,15,17]. The most common side effect noted in 7 out of 10 studies was a burning sensation [10,12,13,14,16,17,18]. The remaining three studies did not report any significant side effects [11,15,19]. Control groups exhibited varying side effects; notably, steroid treatments (methylprednisolone, dexamethasone) significantly increased intraocular pressure (IOP) [16,18].

Overall, tacrolimus groups had fewer serious side effects compared to the control groups in two studies where steroids were used [16,18], while side effects were comparable across groups in seven studies [10,11,12,14,15,17,19]. In one study comparing tacrolimus to sodium cromoglycate, tacrolimus caused a burning sensation, whereas sodium cromoglycate had no side effects [13].

As with any systematic review, our study is not without its limitations. Despite a rigorous methodology and comprehensive data analysis, certain constraints inevitably influenced the scope and interpretation of our findings. Acknowledging these limitations is essential for a balanced understanding of the review’s conclusions and for identifying areas where future research could provide further insights or improvements. The limitations discussed herein pertain to aspects of study selection, data heterogeneity, methodological quality, and other relevant factors that may impact the robustness of the evidence presented.

A key limitation of this systematic review lies in the potential exclusion of relevant studies due to the restrictive search strategy employed. While comprehensive searches were conducted in major databases such as PubMed and Cochrane, the omission of grey literature, conference proceedings, and studies published in languages other than English may have led to an incomplete dataset. This exclusion could have resulted in a selection bias, potentially overlooking studies with valuable insights or alternative perspectives on the topical application of tacrolimus in ophthalmology. Furthermore, the specificity of the search terms used, without the inclusion of broader synonyms or alternative keywords, may have limited the capture of studies addressing similar uses of tacrolimus but under different terminologies or within broader treatment contexts.

The relatively short follow-up periods reported in the included randomized controlled trials (RCTs) also present a significant limitation. Although some studies lasted for up to 12 months, the majority of trials had shorter follow-up durations, often ranging from 1 month to less than a year. This limited observation period restricts the ability to fully assess long-term efficacy, recurrence rates, and delayed adverse effects associated with the use of topical tacrolimus in ophthalmic conditions. Given the chronic and recurrent nature of many ophthalmic disorders, particularly those involving immune modulation, the absence of long-term data precludes a comprehensive understanding of the sustained safety and therapeutic impact of tacrolimus, potentially skewing the review’s conclusions toward short-term outcomes.

In addition, the generalizability of the findings is constrained by considerable heterogeneity across the studies, including variation in study populations, geographic distribution, and drug formulations. The studies spanned seven countries across four continents, yet differences in patient demographics—such as the younger populations often included in studies on VKC versus the older demographics in studies on rarer conditions—limit the external validity of the results. Additionally, disparities in galenic formulations, like the use of ointments versus eye drops, introduce complexity in comparing clinical outcomes due to potential differences in pharmacokinetics and patient adherence. Variations in the frequency of administration, drug concentrations, and total therapy duration further complicate direct comparisons. This issue is further complicated by inconsistent outcome measures, which make it challenging to synthesize findings across studies and preclude making unified conclusions about tacrolimus’s efficacy in diverse clinical contexts. Future research in larger, more diverse cohorts with standardized endpoints is essential to confirm and expand on these results, ensuring broader applicability to various ophthalmic conditions and patient populations.

Another notable limitation lies in the moderate risk of bias present in some included studies. While these biases were not severe enough to categorize the studies as high-bias trials, they nonetheless raise concerns about methodological rigor. Specifically, several studies lacked sufficient detail on essential procedures such as randomization, blinding, and data completeness, which could introduce selection, performance, and detection biases. These issues, while not disqualifying, still potentially affect the studies’ internal validity and the reliability of their reported outcomes. The moderate bias in these trials may have influenced their findings, especially regarding treatment efficacy and adverse event rates, thereby impacting the robustness of the overall conclusions drawn from the pooled data in this review.

Lastly, while this review includes studies published up to March 2025, it was limited by the absence of randomized controlled trials between 2022 and 2024 that met the review’s inclusion and exclusion criteria and passed the bias assessment. Consequently, the findings reflect all pertinent research within the defined temporal scope, though ongoing innovations in ophthalmic treatments beyond this period may yield new insights.

It is evident that more RCTs on the topical use of tacrolimus in ophthalmology are needed. These trials should be well-organized, double-blinded, and free from bias. Methods for measuring outcomes must be rigorously planned and documented. It is crucial to include less commonly studied outcomes, such as quality-of-life assessments or subjective impressions from clinicians or patients (self-assessment), in future trials. High-quality studies should transparently report methods (randomization, blinding, allocation concealment, ethics, outcomes measured) and results. Proper statistical analyses should be employed to address patient dropout. Less researched diseases, like ocular GVHD or dry eye due to Sjögren syndrome, should be prioritized in these trials. Furthermore, systematic reviews and meta-analyses are crucial for generating statistically significant results that can guide evidence-based medical practices for clinical ophthalmologists.

## 5. Conclusions

The review of the existing literature demonstrates that topical tacrolimus (TAC) is efficacious in the treatment and management of various ocular surface diseases. It has shown effectiveness both when compared to other medications and when used alone across different conditions. Tacrolimus significantly improved most measured outcomes, indicating its potential as a valuable therapeutic option in ophthalmology. While the efficacy of tacrolimus is well-supported by the literature, adverse reactions observed in studies were generally mild, with the most common being a burning sensation upon application. Importantly, none of these adverse effects were deemed severe or dangerous, highlighting the overall tolerability of tacrolimus in clinical settings. However, it is notable that the number of trials, systematic reviews, and meta-analyses investigating the topical use of tacrolimus in ophthalmology remains limited. This underscores the need for further research to substantiate and expand upon the qualitative and quantitative findings presented in this review. Future studies should aim for rigorous methodologies, including well-designed randomized controlled trials (RCTs) with robust sample sizes and standardized outcome measures. By conducting more comprehensive research, we can validate the effectiveness and safety profile of tacrolimus more conclusively. This will not only enhance our understanding of its therapeutic potential across different ocular diseases but also inform clinical practice guidelines, ultimately improving patient outcomes in ophthalmic care.

## Figures and Tables

**Table 1:** Demographic data of studies included in the systematic review.

Author/Year	Country	Study Type	Patient Size	Age (T/C)	Condition
Pucci N/2015 [10]	Italy	RCT cros	30/30 + 25/25 (part2) (eyes of total 30 and later 25 patients)	9.05 ± 2.12	Cyclosporine-resistant VKC
Zanjani H/2017 [11]	Iran	RCT	20/20	10.9 ± 3.4/ 11.3 ± 5.4	Resistant VKC
Müller GG/2014 [12]	Brazil	RCT pros	11/10	10.8 ± 5.2/10 ± 2.8	Refractory VKC
Müller EG/2017 [13]	Brazil	RCT pros	8/8	10.8 ± 2.4/12.5 ± 2.3	VKC
Labcharoenwongs P/2012 [14]	Thailand	RCT pros	12/12	10.14 ± 2.60/9.07 ± 2.50	VKC
Moawad P/2021 [15]	Egypt	RCT	30/30	49.4 ± 12.92/44.9 ± 12.58	Dry eye secondary to Sjögren syndrome
Abud BT/2016 [16]	USA	RCT pros	24/16	54 ± 12/58 ± 11	Ocular GVHD
Elhamaky RT/2020 [17]	Egypt	RCT pros	21/22/20 (eyes), 12/12/11 (patients)	36 ± 11.2/36.2 ± 10.2/38.2 ± 10.2	Adenoviral corneal SEIs
Bhargava R/2019 [18]	India	RCT	40/40	26 ± 4.1/25.4 ± 3.7	Adenoviral corneal SEIs
Hashemian NM/2017 [19]	Iran	RCT pros	17/14 (eyes and patients)	46.3 ± 20.5/36.3 ± 15.7	Acute endothelial graft rejection after PKP

Table notes: T/C = Tacrolimus/Control; RCT = Randomized Controlled Trial; VKC = vernal keratoconjunctivitis; SEIs = subepithelial infiltrates; GVHD = graft-versus-host disease; PKP = Penetrating Keratoplasty; Pros = Prospective; Cros = Crossover.

**Table 2:** Clinical data and results of studies included in the systematic review.

Author/Year	Interventions (T/C)	Drug Conc. (T/C)	Galenic (T/C)	Frequency of Adminis. (T/C)	Duration	Re-Evaluations	Outcome Measures
Pucci N/2015 [10]	Tacrolimus/ cyclosporine A	0.1%/1%	e-d/e-d	One drop/time, 3 times daily	3 (+1) + 3 W (2 parts)	0 W, 3 W, 7 W	Signs, symptoms, quality of life score, side effects
Zanjani H/2017 [11]	Tacrolimus +placebo/ interferon α-2b + placebo	0.005%/ 1,000,000 IU/mL	e-d/e-d	Two drops/time	NA (2 M?)	0 W, 2 W, 1 M, 2 M	Signs, symptoms, side effects
Müller GG/2014 [12]	Tacrolimus + placebo/tacrolimus + olopatadine	0.03%/0.03% + 0.1%	oint/oint + e/d	Twice daily	30 d	0 W, 30 d	Signs, symptoms, clinical impression of the progress, self-assessment, safety, side effects
Müller EG/2017 [13]	Tacrolimus/sodium cromoglycate	0.03%/4%	e-d/e-d	3 times daily	90 d	0 d, 15 d, 30 d, 45 d, 90 d	Signs, symptoms, safety, side effects
Labcharoenwongs P/2012 [14]	Tacrolimus + placebo/cyclosporine + placebo	0.1%/2%	oint/e-d	Twice daily + 4 times daily/ 4 times daily + twice daily	8 W	0 W, 2 W, 4 W, 8 W	Symptoms, signs, side effects
Moawad P/2021 [15]	Tacrolimus +placebo/ cyclosporine +placebo	0.03%/0.05%	e-d/e-d	Twice daily	6 M	0 d, 90 d, 180 d	Symptoms, frequency of artificial tears, TBUT, staining scores, Schirmer, meibum quality and expressibility
Abud BT/2016 [16]	Tacrolimus/methylprednisolone	0.05%/0.5%	e-d/e-d	Twice daily	10 W	0 W, 5 W, 10 W	Side effects, symptoms, staining scores, TBUT, Schirmer, HLA-DR, ICAM-1
Elhamaky RT/2020 [17]	Tacrolimus/Te-PTK with MMC/control group	0.03%/0.02% (MMC)	oint/surgery	Once daily	2–6 M	0 W, 1 W, 1 M, 3 M, 6 M, 12 M	Changes in corneal densitometry, changes in corneal HOA, subjective evaluations of effectiveness, objective evaluations of effectiveness
Bhargava R/2019 [18]	Tacrolimus/Dexamethasone	0.03%/0.05%	oint/oint	Twice daily	6 M	(side effects 1 M, 3 M, 6 M), (IOP 1 M, 2 M, 3 M, 4 M, 5 M, 6 M)	Symptom score, SEI score, VA, IOP, side effects
Hashemian NM/2017 [19]	Tacrolimus +Prednisolone/Placebo+ Prednisolone	0.05%	e-d/e-d	4 times daily until rejection reversal and then 0.01% every 6 h	Until rejection reversal and then for 3 months	1 d, 3 d, 1 w, and every 3 to 5 days until reversal. Then every 2 w until 2 M, monthly for 3 M, and every 3 M thereafter.	Rejection reversal or treatment failure, time to rejection reversal, recurrence of rejection

Table notes: T/C = tacrolimus/control; e-d = eye drops; Oint = Ointment; SEI = subepithelial infiltrates; W = Weeks; M = Months; d = Days; NA = Not available; TBUT = Tear break-up time; Te-PTK = Transepithelial phototherapeutic keratectomy; MMC = Mitomycin C; HOA = Higher-order aberrations; VA = Visual acuity; IOP = Intraocular pressure.

**Table 3:** Key findings of studies included in the systematic review.

Study	Patient Population	Condition Treated	Key Findings
Pucci N et al. (2015) [10]	30 patients	Cyclosporine-resistant VKC	Significant improvement in signs and symptoms
Zanjani H et al. (2017) [11]	40 patients	Resistant VKC	Effective as InterferonA-2b
Muller GG et al. (2014) [12]	21 patients	Refractory VKC	No significant difference with the addition of olopatadine
Muller EG et al. (2017) [13]	16 patients	VKC	Superior to sodium cromoglycate
Labcharoenwongs P et al. (2012) [14]	24 patients	VKC	Effective as cyclosporine
Moawad P et al. (2021) [15]	60 patients	Dry eye secondary to Sjogren syndrome	Improved symptoms and signs similar to cyclosporine
Abud BT et al. (2016) [16]	40 patients	Ocular GVHD	Tacrolimus more effective than methylprednisolone in reducing corneal staining
Elhamaky RT (2020) [17]	35 patients	Adenoviral corneal SEIs	Tacrolimus and Te-PTK both effective in reducing corneal densitometry and aberrations
Bhargava R et al. (2019) [18]	80 patients	Adenoviral corneal SEIs	Tacrolimus more effective than dexamethasone in improving SEI and symptoms
Hashemian NM et al. (2017) [19]	31 patients	Acute endothelial graft rejection after PKP	Tacrolimus with prednisolone significantly reduced time to rejection reversal and recurrence of rejection episodes

Table notes: VKC = vernal keratoconjunctivitis; SEIs = subepithelial infiltrates; GVHD = graft-versus-host disease; PKP = Penetrating Keratoplasty; Te-PTK = transepithelial phototherapeutic keratectomy.

**Table 4:** Side effects.

Study Reference	Condition Treated	Comparison	Side Effects in Tacrolimus Group	Side Effects in Control Group
Pucci N et al. (2015) [10]	Cyclosporine-resistant VKC	TAC 0.1% vs. Cyclosporine	Burning sensation, stinging, pain on administration	Burning sensation, stinging, pain on administration
Zanjani H et al. (2017) [11]	Resistant VKC	TAC 0.005% vs. InterferonA-2b	No significant side effects observed	No significant side effects observed
Muller GG et al. (2014) [12]	Refractory VKC	TAC 0.03% vs. TAC 0.03% + Olopatadine	Burning sensation (in 80% of patients)	Burning sensation (in 80% of patients)
Muller EG et al. (2017) [13]	VKC	TAC 0.03% vs. Sodium cromoglycate	Burning sensation	No significant side effects observed
Labcharoenwongs P et al. (2012) [14]	VKC	TAC 0.1% vs. Cyclosporine	Burning sensation, declining course that became non-significant	Burning sensation, declining course that became non-significant
Moawad P et al. (2021) [15]	Dry eye secondary to Sjogren syndrome	TAC 0.03% vs. Cyclosporine	No significant side effects observed	No significant side effects observed
Abud BT et al. (2016) [16]	Ocular GVHD	TAC 0.05% vs. Methylprednisolone	Burning sensation	Increased IOP
Elhamaky RT (2020) [17]	Adenoviral corneal SEIs	TAC 0.03% vs. Te-PTK with MMC	Burning sensation, foreign body sensation	Burning sensation, foreign body sensation
Bhargava R et al. (2019) [18]	Adenoviral corneal SEIs	TAC 0.03% vs. Dexamethasone	Burning sensation	Increased IOP
Hashemian NM et al. (2017) [19]	Acute endothelial graft rejection after PKP	TAC 0.05% + Prednisolone vs. Prednisolone alone	No significant side effects observed	No significant side effects observed

Table notes: VKC = vernal keratoconjunctivitis; SEIs = subepithelial infiltrates; GVHD = graft-versus-host disease; PKP = Penetrating Keratoplasty; TAC = Tacrolimus; Te-PTK = transepithelial phototherapeutic keratectomy; MMC = Mitomycin C; IOP = intraocular pressure.

## Data Availability

The raw data supporting this study’s findings are available from the corresponding author upon request.

## Supplementary Materials

The following supporting information can be downloaded at: https://www.mdpi.com/article/10.3390/jcm14155347/s1.
