# Supplementary material for: Topical Use of Tacrolimus in Corneal and Ocular Surface Pathologies: A Systematic Review
Source: J Clin Med. 2025 Jul 29;14(15):5347. doi: 10.3390/jcm14155347 (PMC12347582; doi:10.3390/jcm14155347)
Supplement: Supplementary file 1 [file jcm-14-05347-s001.zip › jcm-3754585-supplementary.pdf]

Database	Search Date	Search Fields	Search String
Pubmed	Till 16 March 2025	All Fields	tacrolimus AND (eye OR ophthalmic OR ophthalmology OR ocular OR conjunctivitis OR keratoconjunctivitis OR keratoplasty OR dry eye OR keratitis OR sicca OR uveitis)
Cochrane Library	Till 16 March 2025	All Text	tacrolimus AND (eye OR ophthalmic OR ophthalmology OR ocular OR conjunctivitis OR keratoconjunctivitis OR keratoplasty OR dry eye OR keratitis OR sicca OR uveitis)

Supplementary Table S1.
